# Identification of Potential Osteoporosis miRNA Biomarkers Using Bioinformatics Approaches

**DOI:** 10.1155/2021/3562942

**Published:** 2021-11-02

**Authors:** Wei Lu, Qiang Wang, Yi Xue, Jie Gu, Ping Yao, Yufan Ge, Yiming Miao, Jun Chen

**Affiliations:** Department of Orthopaedics, Changshu Hospital Affiliated to Nanjing University of Chinese Medicine, Changshu 215500, China

## Abstract

Osteoporosis is a degenerative osteoarthropathy commonly found in old people and postmenopausal women. Many studies showed that microRNAs (miRNAs) can regulate the expression of osteoporosis-related genes and are abnormally expressed in patients with osteoporosis. miRNAs therefore have the potential to serve as biomarkers of osteoporosis. In this study, the limma package was used for the differential expression analysis of mRNA expression profiles and 357 significantly differentially expressed genes (DEGs) were obtained. Metascape was used for functional enrichment analysis of DEGs. The result revealed that DEGs were mainly enriched in signaling pathways like MAPK6/MAPK4. Based on the STRING database, the protein-protein interaction (PPI) network of DEGs was constructed. MCODE was used to analyze the functional subsets, and a key functional subset composed of 9 genes was screened out. In addition, the miRNA-mRNA regulatory interaction network (RegIN) was analyzed by the CyTargetLinker plugin, which generated 55 miRNA-mRNA regulatory interactions. Through literature searching, the osteoporosis-related gene FOXO1 in the key functional subset was determined to be the main object of the study. In qRT-PCR assay, the expression of the predicted miRNAs was tested in peripheral blood mononuclear cells of mice with osteoporosis, in which 13 miRNAs were remarkably highly expressed. All in all, this study, based on bioinformatics analysis and testing assay of miRNA expression, determined the potential biomarkers of osteoporosis.

## 1. Introduction

Osteoporosis, characterized by low bone mass and deterioration of the bone architecture, is a systemic skeletal disease resulting in an increased risk of bone brittleness and fracture. It is commonly found in old people and postmenopausal women. Most of the studies pointed out that the dynamic unbalance between osteoblasts and osteoclasts led to bone loss, thus causing osteoporosis [1]. Early prediction of osteoporosis can help the high-risk group avoid fragility fracture [2]. By drawing the receiver operating characteristic (ROC) curve, some studies evaluate the osteoporosis prediction model based on bone mineral density (BMD) and multiple clinical characteristics, and the result revealed bad prediction performance of the model [3]. It is necessary, therefore, to develop more efficient prediction biomarkers for osteoporosis.

As noncoding small RNAs, miRNAs can posttranscriptionally modulate the expression of specific genes, thus affecting various kinds of biological processes [4]. A number of studies on miRNAs affecting osteoporosis exhibited that miRNAs can regulate various genes and inhibit proliferation and differentiation of osteoblasts, thereby causing osteoporosis [5–7]. Meanwhile, works of literature also reported that predictive models for fracture risk due to osteoporosis can be constructed via miRNAs [8]. Hence, the excavation of osteoporosis-related miRNA biomarkers can offer more options for the research and development of osteoporosis biomarkers.

Differentially expressed genes (DEGs) were analyzed and screened out based on gene expression matrices. At the same time, based on multiple bioinformatics analyses and assays, the biomarkers of various diseases can be further screened from DEGs. In the field of osteoporosis study, many studies have used expression matrices for research and screened out key genes through bioinformatics analysis [9–11]. For example, Gong et al. [12] published their studies in 2019, in which they pointed out that ATF2, FBXW7, and RDX play important roles in the occurrence of postmenopausal osteoporosis through enrichment analysis, protein-protein interaction (PPI) network analysis, and the qRT-PCR test, based on DEGs of expression gene microarray in women with postmenopausal osteoporosis and normal females. The studies above showed that bioinformatics analysis based on gene expression matrices, combined with the expression level test, could effectively screen out the key genes associated with osteoporosis.

In this study, we made use of gene expression microarrays of mononuclear cells in women with high and low BMD from the Gene Expression Omnibus (GEO) database. Besides, we performed functional enrichment analysis, PPI network analysis, the miRNA-mRNA regulatory interaction network (RegIN), and the miRNA expression test. Finally, we successfully screened out the potential miRNA biomarkers of osteoporosis.

## 2. Materials and Methods

### 2.1. Expression Profile Data and Study Design

**Figure 1:**
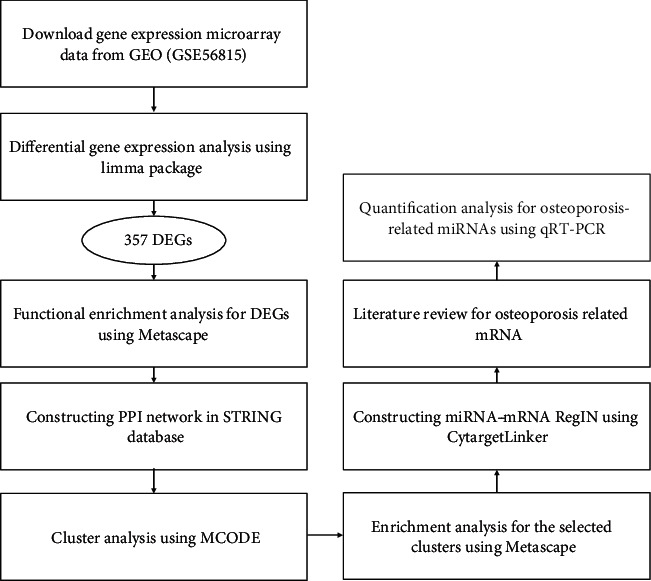
Flowchart of analyses and assays.

Expression profile data (GSE56815) from the GPL96 platform were downloaded from the GEO database (https://www.ncbi.nlm.nih.gov/geo/). The data included the expressed genes of peripheral blood mononuclear cells (PBMC) in 80 Caucasian women who were divided into two groups (high-BMD group: *Z*_BMD_ > +0.84; low-BMD group: *Z*_BMD_ < −0.52) according to the median of *Z*_BMD_ of their femurs: *Z*_BMD_ = (measured bone mineral density − mean bone mineral density of peers of the same race and gender)/standard deviation of bone mineral density of peers of the same race and gender. With 40 women in each group, both of the two groups involved 20 premenopausal women and 20 postmenopausal women. Based on annotation files of the platform, the probe ID was mapped to corresponding gene symbols and the probes without the symbols were removed. A gene symbol was matched with multiple probes whose average expression value was determined to be the final gene expression value. The missing expression data was filled via the *K*-Nearest Neighbor (KNN) [13], followed by log2 scaling. Finally, the limma package [14] was used for standardized processing. Based on the expression profile data, we designed the following bioinformatics analyses and assays (Figure 1).

### 2.2. Differential Expression Analysis and Enrichment Analysis

As 40 women with high BMD were taken as the control group, differential analysis of the expression profile of mononuclear cells in 40 women with low BMD was conducted by using the limma package (FDR < 0.05), and then the DEGs were obtained. Furthermore, the Metascape (http://metascape.org) database [15] was used to perform functional enrichment analysis for key functional subset genes of DEGs and the PPI network and the parameters were set as default.

### 2.3. PPI Network Analysis

With an interaction score > 0.4 as the threshold, the STRING (http://string-db.org/cgi/input.pl) database [16] was used to build a PPI network of DEGs. The MCODE plugin in Cytoscape v3.7.0 was used to select the main functional subsets with high connectivity in the PPI network (parameters were set as Degree = 2, Node score = 0.2, *K*‐core = 2, and Max.depth = 100).

### 2.4. miRNA-mRNA RegIN Analysis

The CyTargetLinker v4.1.0 plugin in Cytoscape v3.7.0 [17] can visualize the RegIN between miRNAs and target genes. Based on miRTarBase v8.0 and TargetScan v7.2 databases, CyTargetLinker v4.1.0 was used to construct miRNA-mRNA RegIN.

### 2.5. Osteoporosis Induction and PBMC Sample Collection

Eight female C57 mice aged 8 weeks were purchased from Charles River Laboratories (Shanghai, China), and they were housed in the cage with food and drink, at room temperature (22 ± 1°C), and with day and night alternation for 12 h/12 h. The mice were randomly divided into the normal group (*n* = 4) and osteoporosis group (*n* = 4). Mice in the osteoporosis group were treated daily with retinoic acid gavage (70 mg/kg retinoic acid, vegetable oil solvent), while the mice in the normal group were treated daily with control solvent gavage (vegetable oil solvent). Fifteen days later, a heparin sodium capillary tube was used to collect 500 *μ*l whole blood from orbits of all of the mice, and the mice would be killed after that. Based on the instructions of the manufacturer, the EasySep™ Mouse Monocyte Isolation Kit (STEMCELL, Canada) was used to extract PBMC from the whole blood collected from the mice. All of the mouse-related assays were approved by the Animal Ethics Committee of Changshu Hospital Affiliated to Nanjing University of Chinese Medicine.

### 2.6. Determination of miRNA Expression in PBMC by qRT-PCR Assay

QIAzol (QIAGEN, USA) was used to extract RNA from PBMC according to the instructions of the manufacturer. Afterwards, reverse transcription was performed by the miRNA Reverse Transcription Kit (QIAGEN, USA) to obtain the corresponding cDNAs. Finally, the miScript SYBR Green PCR Kit (QIAGEN, Canada) was used to perform qRT-PCR (ABI7500, Thermo Fisher Scientific, USA) on obtained cDNAs. U6 served as an internal reference in all PCR reactions. Primers used in the reactions were listed in Supplementary Table 2. Relative expression was calculated by using the 2^-*ΔΔ*Ct^ method. All samples were tested in triplicate.

### 2.7. Data Analysis

GraphPad Prism (GraphPad Software, USA) was used to process the miRNA expression data of the mice. One-way analysis of variance was used for differential comparison between groups. The *t*-test was used for the post hoc test. *p* < 0.05 indicated a significant difference in statistics.

## 3. Results

### 3.1. Differential Expression Analysis and Enrichment Analysis on Osteoporosis-Related DEGs

**Figure 2:**
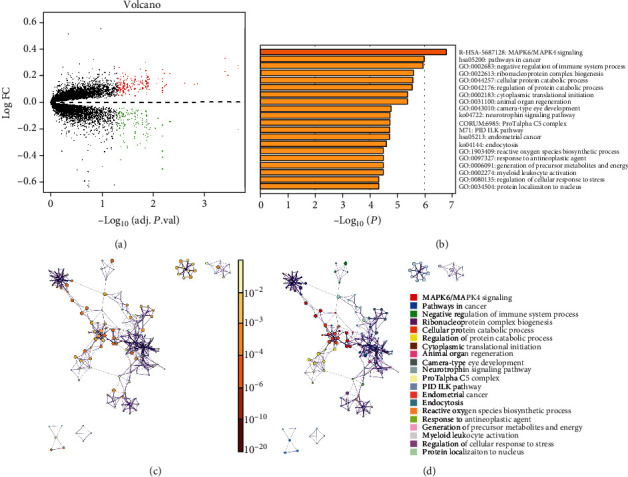
Osteoporosis-related DEGs and enrichment analysis. (a) Volcano plot of osteoporosis-related DEGs. Red represents significantly upregulated genes, while green represents significantly downregulated genes. (b) Bar chart of DEG enrichment analysis. The functions and signaling pathways are ordered according to the *p* value (smaller *p* value indicates higher ranking). (c) Enrichment analysis network based on *p* value. Deeper color means a higher significance of gene enrichment. The bigger the node is, the more genes are included. (d) Enrichment analysis network based on functions and signaling pathways. Each color represents a specific function or signaling pathway, and nodes with the same color belong to the same function or signaling pathway.

PBMC is a cell model suitable for the study of osteoporosis [18]. We therefore studied osteoporosis-related genes based on PBMC gene expression profiles. Firstly, we analyzed the differential expression of the gene profiles of PBMC samples from women in high- and low-BMD groups using the limma package and screened 357 DEGs, including 209 upregulated genes and 148 downregulated genes (Figure 2(a)) (Supplementary Table 1). Based on the 357 DEGs, functional enrichment analysis was performed by Metascape. The enrichment results showed that these genes were mainly enriched in signaling pathways like MAPK6/MAPK4 signaling (Figures 2(b)–2(d)).

### 3.2. PPI Network Analysis

**Figure 3:**
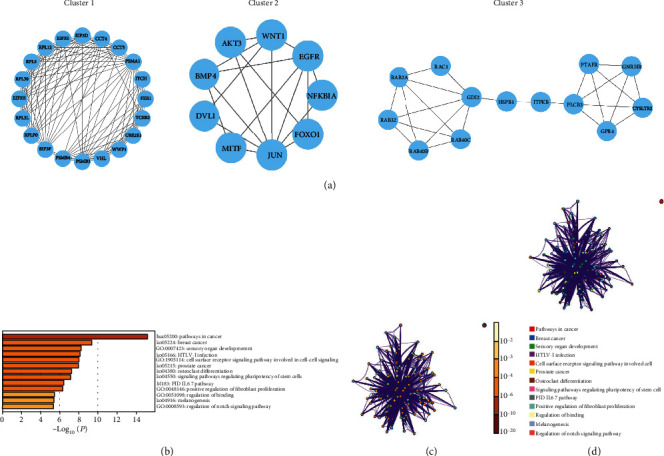
PPI network analysis of DEGs and enrichment analysis of key subsets. (a) Screening of key functional subsets in the PPI network via MCODE. (b) Bar chart of enrichment analysis on genes in Cluster 2. The functions and signaling pathways are ordered according to the *p* value (smaller *p* value means higher ranking). (c) Enrichment analysis network based on *p* value. Deeper color means a higher significance of gene enrichment. The bigger the node is, the more genes are included. (d) Enrichment analysis network based on functions and signaling pathways. Each color represents a specific function or signaling pathway, and nodes with the same color belong to the same function or signaling pathway.

To analyze the corresponding protein regulatory network, a PPI network (286 nodes and 834 lines) was constructed for these DEGs using the STRING database. Subsequently, MCODE was used to screen out the top three functional subsets (Clusters 1, 2, and 3) with the highest score from the PPI network (Figure 3(a)). By consulting the literature, we found that EIF3, RPL3, and RPL30 in Cluster 1 are mostly associated with biological functions such as cell proliferation and cell cycle monitoring [19–21]. FOXO1, BMP4, WNT1, and EGFR in Cluster 2 are related to osteoporosis [22–25]. The genes in Cluster 3 are mostly members of the RAB gene family, which usually encodes GTPase and has a high diversity in functions. For example, RAB2A plays a role in the activation of breast cancer stem cells which induces tumors [26, 27]. Therefore, Cluster 2 was selected for further analysis. We took the genes in Cluster 2 as study objects and performed functional enrichment analysis on them (Figures 3(b)–3(d)). The analysis results demonstrated that these genes were mainly enriched in osteoclast differentiation.

### 3.3. miRNA-mRNA RegIN

**Figure 4:**
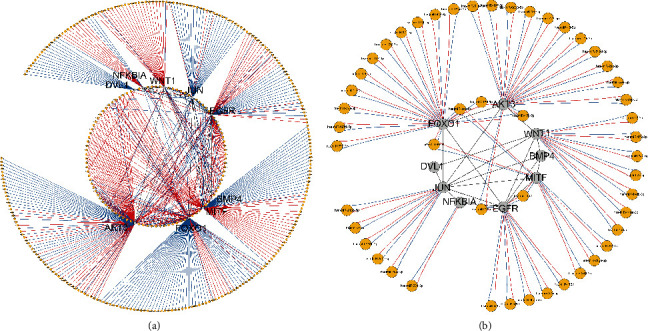
miRNA-mRNA RegIN. (a) miRNA-mRNA RegIN based on two databases. Blue and red represent the miRNA-mRNA regulatory interactions predicted by TargetScan and miRTarBase databases, respectively. (b) RegIN constructed by the common miRNA-mRNA regulatory interactions predicted by miRTarBase and TargetScan databases.

To investigate the miRNAs regulating the genes above, CyTargetLinker was used to predict the RegIN of genes in Cluster 2 and miRNAs. The result showed that a total of 264 miRNAs were predicted from the miRTarBase v8.0 database and 266 miRNAs were predicted from the TargetScan v7.2 database. Besides, a miRNA-mRNA RegIN with 357 nodes and 548 lines was built (Figure 4(a)). To increase the accuracy of the prediction, we screened the common miRNA-mRNA regulatory interactions predicted from the two databases and selected some miRNAs within (Supplementary Table 3) for later experiments (Figure 4(b)).

### 3.4. Screening of Osteoporosis-Related miRNAs

**Figure 5:**
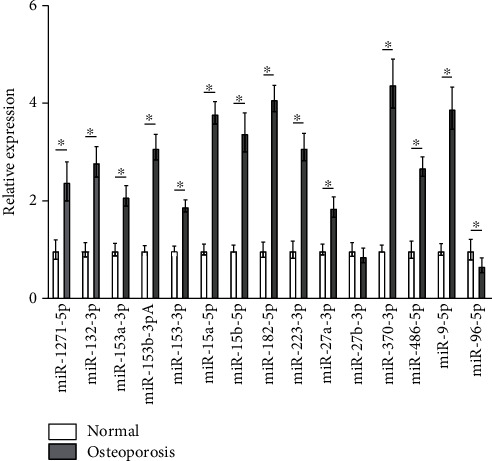
Expression of key miRNAs in mice with osteoporosis. qRT-PCR test of expression of miRNAs regulating FOXO1 predicted in the PBMC of mice with osteoporosis (∗ represents *p* < 0.05).

By searching the literature, we first confirmed that FOXO1 had a strong correlation with osteoporosis. As an important regulatory gene in bone metabolism, FOXO1 modulates osteoblast differentiation and proliferation and osteoclast generation [28]. Meanwhile, many studies revealed that the upregulation of FOXO1 can restore the osteoblast viability of mice with osteoporosis [29–31]. We therefore concluded that FOXO1 expression was negatively correlated with osteoporosis progression. In addition, the relatively accurate miRNA-mRNA RegIN predicted by CyTargetLinker showed that the number of miRNAs regulating FOXO1 was the largest (Figure 4(b)). To further predict the expression of the obtained miRNAs regulating FOXO1 in mice with osteoporosis, we firstly constructed a model of mice with osteoporosis. Then, we used qRT-PCR to test the expression of miRNAs regulating FOXO1 predicted in PBMC of mice with osteoporosis and normal mice (Figure 5). The result showed that miR-96-5p was significantly lowly expressed in the osteoporosis group compared to the normal group, and miR-27b-3p expression had no significant difference between the two groups. Apart from the 2 miRNAs above, all of the rest 13 miRNAs were markedly highly expressed in the osteoporosis group.

## 4. Discussion

Conventional osteoporosis-related markers are mostly protein biomarkers, such as bone formation-related serum total osteocalcin, bone alkaline phosphatase (ALP), type I procollagen N-terminal propeptide (PINP) and bone resorption-related type I collagen cross-linked C-terminal peptide, and serum type I collagen cross-linked N-terminal peptide (S-NTX) [32]. Although these biomarkers, to some extent, can reflect bone metabolism, they have some inadequacies. For instance, (i) the test results of these biomarkers will be affected by the different clinical characteristics of patients [33]; (ii) the results of these biomarkers tested in different laboratories may be greatly different [34]; and (iii) protein, when used as biomarkers to send diagnostic messages, may be less efficient than miRNAs [32]. Thus, miRNAs are hopefully to serve as novel osteoporosis-related biomarkers and make up for the deficiencies of conventional biomarkers. Based on bioinformatics analyses and experiments, the study determined potential miRNA biomarkers related to osteoporosis.

Functional enrichment analysis showed that DEGs were mainly gathered in MAPK6/MAPK4 signaling. Stimulated by various signaling molecules, the MAPK signaling pathway regulates multiple cell functions, including proliferation, differentiation, mitosis, and apoptosis [35]. Moreover, a study revealed that the MAPK signaling pathway is also involved in the proliferation of osteoclasts [36]. In addition, studies exhibited that the traditional Chinese medicine- (TCM-) mediated MAPK signaling pathway modulates bone metabolism in osteoporosis models treated with TCM *in vivo* and *in vitro* [37–39]. In summary, it could be concluded that the results of enrichment analysis on DEGs in this study were consistent with the results of various studies.

FOXO1 was selected from PPI network Cluster 2 of DEGs as a key gene to analyze the upstream miRNAs. In fact, as a member of the FOXO family (including FOXO1, FOXO3, FOXO4, and FOXO6), FOXO1 plays a significant role in bone metabolism. A great number of experiments proved that FOXO1 can activate the proliferation and differentiation of osteoblasts and suppress the differentiation and viability of osteoclasts at the same time [28]. As a major regulator of oxide balance and physiological metabolism of bone cells, FOXO1 provides a favorable intracellular environment for bone cell function by resisting the adverse effects of oxidative stress [40]. A study has shown that activation of the SIRT1/FOXO1 signaling pathway can promote bone formation [29]. FOXO1 therefore plays an important role in the bone metabolism regulation of osteoporosis. Based on this conclusion, we speculated that osteoporosis-related miRNA biomarkers could be found in the miRNAs regulating FOXO1.

Based on the significance of FOXO1 in osteoporosis, we screened out 15 possible miRNAs regulating FOXO1 in miRNA-mRNA RegIN. To test the expression of these miRNAs in mice with osteoporosis, we firstly constructed models of mice with osteoporosis and then carried out the test by qRT-PCR. The result showed that 13 miRNAs were remarkably highly expressed in PBMC of mice with osteoporosis. Among the 13 miRNAs, the expression trends of miR-1271-5p, miR-135a-5p, miR-135b-5p, miR-153-3p, miR-223-3p, miR-27a-3p, miR-370-3p, and miR-9-5p in the current study were in line with their trends in various studies [5, 7, 41–46]. Our result revealed that the expression of the 13 miRNAs was positively correlated with the progression of osteoporosis. Hence, it could be speculated that the 13 miRNAs above could serve as the potential biomarkers of osteoporosis.

In short, this study predicted the potential miRNA biomarkers of osteoporosis via differential analysis of gene expression, functional enrichment analysis, PPI network analysis, and miRNA-mRNA RegIN analysis, and the possibility of the selected miRNAs being biomarkers of osteoporosis was verified by qRT-PCR. Additionally, we found that there is a certain correlation between female osteoporosis and breast cancer in physiological metabolism. However, there are no reports about the linkage of the two diseases in the gene drive, which will be further explored in our future research. Our experiment results revealed the potential of miRNAs being biomarkers of osteoporosis to some degree, but it was not perfect enough. For instance, this study was a retrospective study. Due to limited conditions, the experimental samples in this study were peripheral blood samples from mouse models with osteoporosis. In the future, we will further verify the conclusions of this study by collecting peripheral blood samples from clinical patients. For another, this study did not further study the interactions between these miRNAs and target mRNAs based on the selected miRNAs. In a future study, we plan to design a series of experiments for the screened-out miRNAs so as to verify the binding relationships between these miRNAs and FOXO1 at the molecular level and the regulatory functions of the miRNA-mRNA regulatory axis at the cellular and animal levels.

## Data Availability

The data used to support the findings of this study are included within the article. The data and materials in the current study are available from the corresponding authors on reasonable request.

## Supplementary Materials

Supplementary 1 Supplementary Table 1: differential expression analysis on PBMC expression profiles in women with high and low BMD.

Supplementary 2 Supplementary Table 2: primer sequences of qRT-PCR.

Supplementary 3 Supplementary Table 3: miRNAs regulating genes in key functional subsets.

## References

[1] Lane J. M., Russell L., Khan S. N. (2000). Osteoporosis. *Clinical Orthopaedics and Related Research*.

[2] Lee S. H., Cho E. H., Ahn S. H. (2016). Prediction of future osteoporotic fracture occurrence by genetic profiling: a 6-year follow-up observational study. *The Journal of Clinical Endocrinology and Metabolism*.

[3] Bolland M. J., Siu A. T. Y., Mason B. H. (2011). Evaluation of the FRAX and Garvan fracture risk calculators in older women. *Journal of Bone and Mineral Research*.

[4] Correia de Sousa M., Gjorgjieva M., Dolicka D., Sobolewski C., Foti M. (2019). Deciphering miRNAs' action through miRNA editing. *International Journal of Molecular Sciences*.

[5] Yang Q., Zhou Y., Wang T. (2021). miRNA-1271-5p regulates osteogenic differentiation of human bone marrow-derived mesenchymal stem cells by targeting forkhead box O1 (FOXO1). *Cell Biology International*.

[6] Qu X., Chen Z., Fan D., Sun C., Zeng Y. (2016). miR-132-3p regulates the osteogenic differentiation of thoracic ligamentum flavum cells by inhibiting multiple osteogenesis-related genes. *International Journal of Molecular Sciences*.

[7] Chen B., Yang W., Zhao H. (2019). Abnormal expression of miR-135b-5p in bone tissue of patients with osteoporosis and its role and mechanism in osteoporosis progression. *Experimental and Therapeutic Medicine*.

[8] Tang X., Bai Y., Zhang Z., Lu J. (2020). A validated miRNA signature for the diagnosis of osteoporosis related fractures using SVM algorithm classification. *Experimental and Therapeutic Medicine*.

[9] Yan B., Li J., Zhang L. (2015). Identification of B cells participated in the mechanism of postmenopausal women osteoporosis using microarray analysis. *International Journal of Clinical and Experimental Medicine*.

[10] Liu Y., Wang Y., Yang N., Wu S., Lv Y., Xu L. (2015). In silico analysis of the molecular mechanism of postmenopausal osteoporosis. *Molecular Medicine Reports*.

[11] Ma M., Luo S., Zhou W. (2017). Bioinformatics analysis of gene expression profiles in B cells of postmenopausal osteoporosis patients. *Taiwanese Journal of Obstetrics & Gynecology*.

[12] Gong R., Ren S., Chen M. (2019). Bioinformatics analysis reveals the altered gene expression of patients with postmenopausal osteoporosis using Liuweidihuang pills treatment. *BioMed Research International*.

[13] Troyanskaya O., Cantor M., Sherlock G. (2001). Missing value estimation methods for DNA microarrays. *Bioinformatics*.

[14] Ritchie M. E., Phipson B., Wu D. (2015). *limma* powers differential expression analyses for RNA-sequencing and microarray studies. *Nucleic Acids Research*.

[15] Zhou Y., Zhou B., Pache L. (2019). Metascape provides a biologist-oriented resource for the analysis of systems-level datasets. *Nature Communications*.

[16] Szklarczyk D., Gable A. L., Lyon D. (2019). STRING v11: protein-protein association networks with increased coverage, supporting functional discovery in genome-wide experimental datasets. *Nucleic Acids Research*.

[17] Shannon P., Andrew M., Owen O. (2003). Cytoscape: a software environment for integrated models of biomolecular interaction networks. *Genome Research*.

[18] Zhou Y., Deng H. W., Shen H. (2015). Circulating monocytes: an appropriate model for bone-related study. *Osteoporosis International*.

[19] Lee A. S., Kranzusch P. J., Cate J. H. (2015). eIF3 targets cell-proliferation messenger RNAs for translational activation or repression. *Nature*.

[20] Wang C. H., Wang L. K., Wu C. C. (2019). The ribosomal protein RPLP0 mediates PLAAT4-induced cell cycle arrest and cell apoptosis. *Cell Biochemistry and Biophysics*.

[21] Russo A., Esposito D., Catillo M., Pietropaolo C., Crescenzi E., Russo G. (2013). Human rpL3 induces G_1_/S arrest or apoptosis by modulating p21^waf1/cip1^ levels in a p53-independent manner. *Cell Cycle*.

[22] Rached M. T., Kode A., Xu L. (2010). FoxO1 is a positive regulator of bone formation by favoring protein synthesis and resistance to oxidative stress in osteoblasts. *Cell Metabolism*.

[23] Chen S., Jia L., Zhang S., Zheng Y., Zhou Y. (2018). DEPTOR regulates osteogenic differentiation via inhibiting MEG3-mediated activation of BMP4 signaling and is involved in osteoporosis. *Stem Cell Research & Therapy*.

[24] Mäkitie R. E., Kämpe A., Costantini A., Alm J. J., Magnusson P., Mäkitie O. (2020). Biomarkers in WNT1 and PLS3 osteoporosis: altered concentrations of DKK1 and FGF23. *Journal of Bone and Mineral Research*.

[25] Liu G., Xie Y., Su J. (2019). The role of EGFR signaling in age-related osteoporosis in mouse cortical bone. *The FASEB Journal*.

[26] Zhao F., Zhong M., Pei W., Tian B., Cai Y. (2020). miR-376c-3p modulates the properties of breast cancer stem cells by targeting RAB2A. *Experimental and Therapeutic Medicine*.

[27] Luo M. L., Gong C., Chen C. H. (2015). The Rab2A GTPase promotes breast cancer stem cells and tumorigenesis via Erk signaling activation. *Cell Reports*.

[28] Ma X., Su P., Yin C. (2020). The roles of FoxO transcription factors in regulation of bone cells function. *International Journal of Molecular Sciences*.

[29] Jiang Y., Luo W., Wang B., Wang X., Gong P., Xiong Y. (2020). Resveratrol promotes osteogenesis via activating SIRT1/FoxO1 pathway in osteoporosis mice. *Life Sciences*.

[30] Feng Y. L., Jiang X. T., Ma F. F., Han J., Tang X. L. (2018). Resveratrol prevents osteoporosis by upregulating FoxO1 transcriptional activity. *International Journal of Molecular Medicine*.

[31] Ameen O., Yassien R. I., Naguib Y. M. (2020). Activation of FoxO1/SIRT1/RANKL/OPG pathway may underlie the therapeutic effects of resveratrol on aging-dependent male osteoporosis. *BMC Musculoskeletal Disorders*.

[32] Garnero P. (2017). The utility of biomarkers in osteoporosis management. *Molecular Diagnosis & Therapy*.

[33] Hannon R., Eastell R. (2000). Preanalytical variability of biochemical markers of bone turnover. *Osteoporosis International*.

[34] Seibel M. J., Lang M., Geilenkeuser W. J. (2001). Interlaboratory variation of biochemical markers of bone turnover. *Clinical Chemistry*.

[35] Sun Y., Liu W. Z., Liu T., Feng X., Yang N., Zhou H. F. (2015). Signaling pathway of MAPK/ERK in cell proliferation, differentiation, migration, senescence and apoptosis. *Journal of Receptor and Signal Transduction Research*.

[36] Noh J. Y., Yang Y., Jung H. (2020). Molecular mechanisms and emerging therapeutics for osteoporosis. *International Journal of Molecular Sciences*.

[37] Lin J., Zhu J., Wang Y. (2017). Chinese single herbs and active ingredients for postmenopausal osteoporosis: from preclinical evidence to action mechanism. *Bioscience Trends*.

[38] An J., Yang H., Zhang Q. (2016). Natural products for treatment of osteoporosis: the effects and mechanisms on promoting osteoblast-mediated bone formation. *Life Sciences*.

[39] Zhang N. D., Han T., Huang B. K. (2016). Traditional Chinese medicine formulas for the treatment of osteoporosis: implication for antiosteoporotic drug discovery. *Journal of Ethnopharmacology*.

[40] Zhang Y., Xiong Y., Zhou J., Xin N., Zhu Z., Wu Y. (2018). FoxO1 expression in osteoblasts modulates bone formation through resistance to oxidative stress in mice. *Biochemical and Biophysical Research Communications*.

[41] Shi X., Zhang Z. (2019). MicroRNA-135a-5p is involved in osteoporosis progression through regulation of osteogenic differentiation by targeting RUNX2. *Experimental and Therapeutic Medicine*.

[42] Jiang H., Jia P. (2021). miR-153-3p inhibits osteogenic differentiation of periodontal ligament stem cells through KDM6A-induced demethylation of H3K27me3. *Journal of Periodontal Research*.

[43] Long C., Cen S., Zhong Z., Zhou C., Zhong G. (2021). FOXO3 is targeted by miR-223-3p and promotes osteogenic differentiation of bone marrow mesenchymal stem cells by enhancing autophagy. *Human Cell*.

[44] Xu Y., Li D., Zhu Z. (2020). miR‑27a‑3p negatively regulates osteogenic differentiation of MC3T3‑E1 preosteoblasts by targeting osterix. *Molecular Medicine Reports*.

[45] Jia B., Wang Z., Sun X., Chen J., Zhao J., Qiu X. (2019). Long noncoding RNA LINC00707 sponges miR-370-3p to promote osteogenesis of human bone marrow-derived mesenchymal stem cells through upregulating WNT2B. *Stem Cell Research & Therapy*.

[46] Zheng C., Bai C., Sun Q. (2020). Long noncoding RNA XIST regulates osteogenic differentiation of human bone marrow mesenchymal stem cells by targeting miR-9-5p. *Mechanisms of Development*.

