# Supplementary material for: Identification of Potential Osteoporosis miRNA Biomarkers Using Bioinformatics Approaches
Source: Comput Math Methods Med. 2021 Nov 2;2021:3562942. doi: 10.1155/2021/3562942 (PMC8579105; doi:10.1155/2021/3562942)
Supplement: Supplementary 1 — Supplementary Table 1: differential expression analysis on PBMC expression profiles in women with high and low BMD. [file 3562942.f1.pdf]

ID	logFC	AveExpr	t	P.Value	adj.P.Val	B
RAB2A	-0.20751	7.743602	-6.15735	2.70E-08	0.000224	8.684634
VPS35	0.255939	10.5578	6.001193	5.26E-08	0.000224	8.073276
FOXO3	0.27449	9.042238	5.996645	5.37E-08	0.000224	8.055552
TRIM44	0.201728	8.617078	5.82229	1.12E-07	0.000353	7.379525
DPP8	0.330156	8.94489	5.702524	1.86E-07	0.000399	6.9194
POGLUT1	0.230218	7.830905	5.696431	1.91E-07	0.000399	6.896092
SKAP2	0.137709	8.473858	5.366693	7.46E-07	0.001336	5.649775
METTL4	0.133111	6.203244	5.290619	1.02E-06	0.001594	5.366769
TNPO3	0.142207	7.946679	5.157916	1.74E-06	0.002421	4.877513
C15orf49	-0.14475	6.720479	-4.9694	3.68E-06	0.004617	4.192732
NIF3L1	0.215237	8.523611	4.919132	4.49E-06	0.004794	4.012261
GMEB1	-0.1435	7.646675	-4.9136	4.59E-06	0.004794	3.992442
RPL3	0.132579	12.73896	4.854041	5.79E-06	0.005587	3.779983
GLT8D2	0.124023	6.552365	4.824309	6.50E-06	0.005826	3.674418
HLA-DMB	0.217229	11.81829	4.78577	7.55E-06	0.006046	3.538089
MSL3	0.116346	7.923739	4.780396	7.71E-06	0.006046	3.519123
MEX3D	0.075186	5.426001	4.72426	9.58E-06	0.006609	3.321708
Y16709	-0.4183	11.33502	-4.71953	9.76E-06	0.006609	3.305122
HIRA	-0.37346	6.390837	-4.69484	1.07E-05	0.006609	3.218736
DHX35	0.108616	5.880491	4.692659	1.08E-05	0.006609	3.211129
RPL3L	0.091411	5.876289	4.684274	1.12E-05	0.006609	3.181856
FCGR2A	0.246079	10.01319	4.659997	1.23E-05	0.006609	3.097264
ZEB2	-0.50628	7.898739	-4.65654	1.24E-05	0.006609	3.085226
IL17RA	-0.25404	10.1935	-4.63891	1.33E-05	0.006609	3.023998
PAPD7	0.175854	7.974195	4.631942	1.36E-05	0.006609	2.99981
MATN4	-0.16983	6.048397	-4.62361	1.41E-05	0.006609	2.970928
RCAN2	0.086155	5.451943	4.614656	1.46E-05	0.006609	2.939923
NVL	0.155036	6.86589	4.611593	1.47E-05	0.006609	2.929325
THOC2	0.175115	7.96957	4.546584	1.89E-05	0.007958	2.705321
LIMK2	-0.11864	6.228263	-4.54443	1.90E-05	0.007958	2.697946
CBX5	-0.11772	6.579076	-4.52593	2.04E-05	0.008259	2.634539
ADSL	0.141489	9.192967	4.50831	2.18E-05	0.00855	2.574283
UBE2E1	-0.19426	10.44952	-4.48265	2.40E-05	0.00913	2.486769
TBC1D2	-0.24863	6.559715	-4.41449	3.10E-05	0.011432	2.255786
SRP19	-0.19011	9.158361	-4.40473	3.21E-05	0.011516	2.222867
DYNLRB1	-0.15777	9.806531	-4.38569	3.45E-05	0.011893	2.158805
XDH	0.172593	7.239791	4.374498	3.59E-05	0.011893	2.121203
NELFB	-0.20356	7.627986	-4.36875	3.67E-05	0.011893	2.101915
LPIN1	0.095811	6.549144	4.366893	3.70E-05	0.011893	2.095694
ZNF37BP	0.147521	5.517008	4.33823	4.11E-05	0.012689	1.999793
NDUFC1	-0.16247	9.352514	-4.32692	4.28E-05	0.012689	1.962061
ADAM11	0.187196	6.815365	4.325538	4.31E-05	0.012689	1.957448
F7	-0.23827	6.067201	-4.31646	4.45E-05	0.012689	1.927221
SLC25A14	0.117331	7.1443	4.306722	4.62E-05	0.012689	1.894806
PTMA	0.141814	11.02493	4.301823	4.70E-05	0.012689	1.878526
SLC25A5	0.128001	11.51742	4.298941	4.75E-05	0.012689	1.868951
UTP6	0.193938	8.726468	4.288651	4.93E-05	0.012689	1.834801
C1D	-0.25638	8.615928	-4.28846	4.93E-05	0.012689	1.834182
EIF3H	0.171632	10.99127	4.28732	4.96E-05	0.012689	1.830389
PHF20	0.141922	7.459484	4.253354	5.61E-05	0.013288	1.718042
EDA	0.06162	6.076643	4.245435	5.78E-05	0.013288	1.691928
CCT5	0.180452	10.26066	4.235586	5.99E-05	0.013288	1.65949
TRPM6	0.11356	6.310272	4.229958	6.11E-05	0.013288	1.640976
EIF2D	0.152362	8.6916	4.225222	6.22E-05	0.013288	1.625407
CHRNA2	-0.17032	6.575639	-4.21277	6.50E-05	0.013288	1.584515
DHTKD1	0.112053	5.775504	4.210531	6.56E-05	0.013288	1.577182
ARHGEF10	0.072786	5.729392	4.209348	6.59E-05	0.013288	1.573302

CDC42EP3	-0.2318	9.485966	-4.20645	6.65E-05	0.013288	1.563803
SLC29A3	0.152396	8.460927	4.199317	6.83E-05	0.013288	1.540438
IL1R2	0.215547	6.204257	4.199195	6.83E-05	0.013288	1.540042
UBQLN2	-0.26237	8.349032	-4.19174	7.02E-05	0.013288	1.515664
EXOSC9	0.097737	7.590738	4.190908	7.04E-05	0.013288	1.512927
POLR2H	0.158029	8.154057	4.187369	7.13E-05	0.013288	1.50136
STMN4	0.105701	5.604849	4.187042	7.14E-05	0.013288	1.500293
SH3GLB2	-0.2831	6.050321	-4.18619	7.16E-05	0.013288	1.497509
S100A4	-0.10533	12.89468	-4.18407	7.22E-05	0.013288	1.490577
PAF1	-0.14345	7.555722	-4.18294	7.25E-05	0.013288	1.48689
ARID4B	-0.13078	7.276436	-4.18115	7.29E-05	0.013288	1.48104
KDM4B	-0.09589	7.341225	-4.17974	7.33E-05	0.013288	1.476445
DESI2	-0.09478	6.352732	-4.1766	7.41E-05	0.013288	1.466206
UBE2G2	0.150963	7.854436	4.166572	7.69E-05	0.013395	1.433503
GLA	0.186301	8.929913	4.16423	7.75E-05	0.013395	1.425875
LETMD1	0.137577	8.049204	4.162742	7.79E-05	0.013395	1.421028
HIVEP2	-0.09432	5.706616	-4.14881	8.20E-05	0.013664	1.375704
RAB11FIP1	-0.22818	9.137347	-4.14607	8.28E-05	0.013664	1.366827
VHL	-0.07062	4.844463	-4.14116	8.42E-05	0.013664	1.350879
DVL1	-0.19156	6.831801	-4.13761	8.53E-05	0.013664	1.339342
FIG4	0.138528	9.535634	4.135964	8.58E-05	0.013664	1.334017
KLF7	0.200242	8.066012	4.133538	8.66E-05	0.013664	1.326154
FLNC	0.136737	6.682225	4.131766	8.71E-05	0.013664	1.320407
ABCB7	0.158551	7.410488	4.120393	9.08E-05	0.013912	1.283583
GSAP	0.239244	9.650522	4.118194	9.15E-05	0.013912	1.276473
HAPLN2	0.119092	5.896485	4.115605	9.23E-05	0.013912	1.2681
ARHGAP4	-0.06477	6.086603	-4.11315	9.31E-05	0.013912	1.260152
RPL12	0.112392	12.48612	4.104773	9.60E-05	0.014166	1.233115
SNX5	0.166062	9.291558	4.099232	9.79E-05	0.014176	1.215238
WWP1	-0.24854	6.637906	-4.09782	9.84E-05	0.014176	1.210694
ELMO2	-0.14715	8.084069	-4.09488	9.94E-05	0.014176	1.20121
DMTF1	0.30069	8.40935	4.091322	0.000101	0.014196	1.18975
SEC22B	-0.35152	10.17109	-4.08643	0.000102	0.014285	1.174009
SLC25A11	-0.1854	7.846115	-4.07759	0.000106	0.014468	1.145567
CLCA1	0.125926	6.878671	4.076703	0.000106	0.014468	1.142722
PSMD3	-0.24112	6.659917	-4.05159	0.000116	0.01565	1.062197
EIF3D	0.129547	10.48914	4.047471	0.000118	0.015712	1.049009
NPRL2	0.107633	7.303408	4.043707	0.000119	0.015755	1.036973
POLB	0.143215	8.779534	4.039063	0.000121	0.01585	1.022133
ADAM17	-0.15214	7.202081	-4.03199	0.000124	0.016085	0.999548
USP27X	0.1087	5.77064	4.027618	0.000126	0.016121	0.985609
CYP2B7P	-0.12149	7.000215	-4.02515	0.000127	0.016121	0.977758
BRSK2	0.102259	5.624758	4.022747	0.000128	0.016121	0.970086
MANBA	0.162863	9.149358	4.011777	0.000134	0.016351	0.935169
RNF5	-0.13623	6.173704	-4.01014	0.000134	0.016351	0.929963
ATP5C1	0.100274	10.06638	4.007017	0.000136	0.016351	0.920037
BICD2	-0.16582	8.191819	-4.00559	0.000137	0.016351	0.915508
OPA3	-0.12213	5.105722	-4.00492	0.000137	0.016351	0.913382
SMAGP	0.141895	7.03538	3.995669	0.000141	0.016691	0.884007
C6orf48	0.149924	10.07307	3.993718	0.000142	0.016691	0.877821
BAZ2A	-0.08529	7.08599	-3.99095	0.000144	0.016699	0.869057
NIT2	0.194554	7.192714	3.987329	0.000146	0.016758	0.857573
SMCP	0.10619	6.851108	3.964546	0.000158	0.017859	0.785539
MAN2A1	0.249351	9.03144	3.964027	0.000158	0.017859	0.783901
SOAT2	-0.1127	6.451945	-3.95757	0.000162	0.017883	0.763524
PSMB4	0.159272	10.32888	3.956617	0.000162	0.017883	0.760534
DAP3	0.14812	9.423398	3.956034	0.000162	0.017883	0.758696
NFKBIA	-0.30974	11.07148	-3.95077	0.000166	0.018058	0.742107

PYGM	0.098411	5.628853	3.944534	0.000169	0.018297	0.722491
GTF3A	0.132771	10.10289	3.933571	0.000176	0.018849	0.68804
NAP1L2	-0.05921	4.750689	-3.92353	0.000182	0.019355	0.656543
SUPT20H	0.080754	6.666903	3.917561	0.000186	0.019578	0.637845
SAP130	0.162098	7.743414	3.915422	0.000187	0.019578	0.631148
HIPK1	-0.16103	8.909664	-3.9112	0.00019	0.019703	0.617934
EIF3F	0.075446	8.269972	3.907124	0.000193	0.019821	0.605193
MITF	-0.14866	6.759784	-3.90447	0.000195	0.019842	0.596901
MFSD10	-0.31854	6.979855	-3.89712	0.0002	0.020132	0.573956
ZNF236	-0.06741	6.290236	-3.89564	0.000201	0.020132	0.569346
CCT4	0.135877	10.45018	3.890575	0.000204	0.020239	0.553542
CYSLTR2	-0.11231	5.880922	-3.88954	0.000205	0.020239	0.550319
INTS5	-0.09444	6.867608	-3.88336	0.000209	0.020516	0.531061
MSN	-0.15113	11.56592	-3.87281	0.000217	0.021114	0.498259
THRA	-0.06098	6.191362	-3.86377	0.000224	0.021446	0.470191
ABR	-0.18793	7.55379	-3.86277	0.000225	0.021446	0.467095
TTC27	0.156714	6.48036	3.861649	0.000226	0.021446	0.463613
ITPKB	-0.16288	6.970274	-3.85253	0.000233	0.021965	0.435349
FLT3LG	-0.11291	5.518941	-3.84647	0.000238	0.022262	0.416584
ACAA2	0.102713	7.677309	3.840855	0.000242	0.022413	0.399242
ARL4C	0.1771	6.816494	3.835784	0.000247	0.022413	0.383579
TMEM126I	0.196453	9.259727	3.835074	0.000247	0.022413	0.381386
ACTN2	0.049501	5.971254	3.834256	0.000248	0.022413	0.378861
NPY	0.103014	6.146902	3.833863	0.000248	0.022413	0.377648
FRAT2	-0.21766	9.499618	-3.82761	0.000254	0.022487	0.358356
HAMP	-0.22919	5.889421	-3.8273	0.000254	0.022487	0.357396
JUN	-0.10419	5.649393	-3.82598	0.000255	0.022487	0.353345
PTAFR	0.168264	9.185743	3.824643	0.000256	0.022487	0.349217
LASP1	-0.12695	10.26747	-3.81947	0.000261	0.022507	0.33328
HCN2	-0.07289	5.697495	-3.81904	0.000261	0.022507	0.33197
TSNAX	-0.23222	8.700902	-3.81642	0.000264	0.022507	0.323889
TTTY15	-0.07459	5.353289	-3.81629	0.000264	0.022507	0.323513
RPL30	-0.08299	13.35114	-3.81437	0.000266	0.022507	0.317587
FAM129A	-0.33755	8.403508	-3.81215	0.000268	0.022527	0.310758
WT1-AS	0.098055	5.983664	3.806905	0.000272	0.022782	0.294648
KCNF1	-0.26821	6.269309	-3.80324	0.000276	0.022917	0.283409
SLC2A3	-0.25695	8.369089	-3.79884	0.00028	0.02304	0.269891
LRRC23	0.152111	6.298813	3.797831	0.000281	0.02304	0.2668
COMMD3	0.129688	9.378755	3.792912	0.000286	0.023278	0.251721
SLC25A3	0.09963	11.60416	3.788409	0.00029	0.023291	0.237931
AC003989.	0.102661	6.091608	3.787225	0.000291	0.023291	0.234307
BOLA1	0.095198	5.779883	3.787109	0.000291	0.023291	0.233951
RP2	-0.27145	8.283676	-3.7841	0.000294	0.023382	0.224738
FAM216A	-0.17565	6.603982	-3.78191	0.000297	0.0234	0.21806
POFUT1	-0.08217	5.787591	-3.78007	0.000299	0.0234	0.212421
WNT1	0.104002	5.363134	3.778165	0.0003	0.0234	0.206598
RTCB	0.158758	9.122307	3.776548	0.000302	0.0234	0.201659
PCSK1N	0.105318	6.539318	3.772902	0.000306	0.023547	0.190524
GDI2	0.135358	11.50087	3.762861	0.000317	0.024135	0.159898
ZNF787	-0.15639	6.580676	-3.76207	0.000317	0.024135	0.157488
CTC-425F1	-0.33762	8.061371	-3.75993	0.00032	0.024165	0.150971
DNMBP	0.14063	7.323967	3.75749	0.000322	0.02422	0.143539
C21orf59	0.126272	8.160363	3.746495	0.000335	0.024992	0.1101
FABP3	-0.06809	5.762052	-3.74317	0.000338	0.025125	0.100014
SLC6A12	0.119311	6.612222	3.734936	0.000348	0.025684	0.075019
CNOT7	0.142451	9.574049	3.732195	0.000351	0.025772	0.066712
NBPF1	0.166469	10.43923	3.728122	0.000356	0.025978	0.054375
VCP	-0.12154	8.502037	-3.72568	0.000359	0.026003	0.046987

MAGEB4	-0.05108	5.197157	-3.72361	0.000362	0.026003	0.040713
SLC7A2	-0.08408	5.424738	-3.72208	0.000363	0.026003	0.036092
SLC41A3	0.140364	8.025901	3.721032	0.000365	0.026003	0.03292
LMAN2L	0.099788	8.316064	3.712667	0.000375	0.026483	0.007646
RAC1	0.180045	10.75107	3.712273	0.000376	0.026483	0.006456
NUDT6	-0.08114	5.008233	-3.70253	0.000388	0.027214	-0.02294
THEG	0.114354	7.23977	3.693915	0.0004	0.027859	-0.04887
TCEB2	0.106255	9.492133	3.688052	0.000408	0.028257	-0.0665
IRF8	0.176069	11.08871	3.685647	0.000411	0.028329	-0.07373
SRSF8	0.122659	8.548367	3.674769	0.000426	0.029207	-0.10638
NCAN	-0.09284	6.030379	-3.6733	0.000428	0.029207	-0.11077
GPR116	0.059572	5.666179	3.670818	0.000432	0.029291	-0.11821
SREK1IP1	-0.12572	5.696524	-3.66501	0.00044	0.029706	-0.1356
ZIC1	0.085708	5.40726	3.662438	0.000444	0.029803	-0.1433
KCNC1	0.095261	6.288457	3.659736	0.000448	0.029913	-0.15138
PLG	0.09296	5.791596	3.65501	0.000455	0.030229	-0.1655
PAGE4	-0.08359	5.796037	-3.64811	0.000466	0.030771	-0.18609
RAB28	-0.07948	5.680926	-3.64546	0.00047	0.030881	-0.19398
HMGCL	0.129541	8.608719	3.637036	0.000484	0.031462	-0.21908
DCLRE1C	0.182803	7.492517	3.635266	0.000486	0.031462	-0.22435
HSPB1	-0.34857	6.804692	-3.6349	0.000487	0.031462	-0.22545
TUBA8	-0.13242	5.893522	-3.63202	0.000492	0.031462	-0.23401
FLT3	0.195688	7.351215	3.631236	0.000493	0.031462	-0.23634
ATRAID	0.123127	9.385641	3.629868	0.000495	0.031462	-0.2404
ZRSR2	0.111952	8.394922	3.628166	0.000498	0.031462	-0.24546
SMC1A	-0.09164	7.586978	-3.62756	0.000499	0.031462	-0.24726
RHOF	0.134725	7.834852	3.625528	0.000502	0.031517	-0.2533
PSMA7	0.174595	8.687915	3.622942	0.000507	0.031631	-0.26097
ABCD4	0.08669	7.106427	3.614762	0.000521	0.031942	-0.28523
GATAD2A	-0.13083	6.775548	-3.61469	0.000521	0.031942	-0.28545
GCHFR	0.128385	6.757149	3.61458	0.000521	0.031942	-0.28577
GRIN2B	-0.06124	5.794496	-3.61406	0.000522	0.031942	-0.28732
KIZ	0.137208	6.389289	3.611557	0.000526	0.032052	-0.29473
LOC10050	-0.32802	5.968338	-3.60715	0.000534	0.032367	-0.30776
RS1	-0.05886	5.442136	-3.60174	0.000544	0.032795	-0.32379
DHX15	0.133673	10.36526	3.600093	0.000547	0.032795	-0.32864
PSMA1	0.116622	10.82614	3.598839	0.000549	0.032795	-0.33235
CDH16	0.114092	6.180613	3.595244	0.000556	0.033031	-0.34296
IFT140	-0.07735	4.970722	-3.59101	0.000563	0.033339	-0.35547
ZNF7	0.106072	6.022504	3.588938	0.000567	0.03341	-0.36157
ASTE1	0.167035	6.587543	3.585104	0.000574	0.033574	-0.37287
ITCH	0.106904	6.805307	3.584625	0.000575	0.033574	-0.37428
INTS8	0.208893	9.062036	3.574875	0.000594	0.034511	-0.40297
GH1	0.064577	6.464008	3.573149	0.000598	0.034548	-0.40805
SLC22A7	-0.09956	6.007901	-3.56968	0.000604	0.034785	-0.41824
MIA3	0.098491	7.282626	3.562847	0.000618	0.035414	-0.4383
YTHDF2	-0.16791	9.197759	-3.5593	0.000625	0.035668	-0.44871
IGBP1	0.125633	10.01499	3.552606	0.000639	0.036125	-0.4683
EBLN2	0.145969	6.830273	3.551907	0.000641	0.036125	-0.47035
MPZ	0.10501	6.80969	3.550764	0.000643	0.036125	-0.4737
TMEM258	-0.17537	10.45607	-3.5473	0.000651	0.036125	-0.48383
IKZF1	-0.10861	7.329126	-3.54692	0.000651	0.036125	-0.48495
ABO	-0.30071	6.094148	-3.54652	0.000652	0.036125	-0.48609
RP11-414H	0.095684	6.277725	3.545877	0.000654	0.036125	-0.48799
HIST1H2BC	0.075647	5.038746	3.543872	0.000658	0.036204	-0.49385
LST1	0.136881	11.31202	3.539238	0.000668	0.036348	-0.50738
EPB41L4A	0.075912	5.672429	3.538862	0.000669	0.036348	-0.50848
IDO1	0.164837	7.395096	3.538671	0.000669	0.036348	-0.50904

RAB40C	-0.16507	6.400666	-3.53392	0.00068	0.036536	-0.5229
VPS37C	0.121311	8.821191	3.533324	0.000681	0.036536	-0.52464
SLC6A13	0.140773	7.783287	3.532387	0.000683	0.036536	-0.52737
NUP50	0.142267	7.781736	3.531857	0.000684	0.036536	-0.52891
NF2	-0.04363	5.563532	-3.52307	0.000704	0.037441	-0.55449
FOXO1	-0.11622	7.434748	3.51911	0.000713	0.037769	-0.56602
TMBIM6	0.117019	10.93161	3.516189	0.00072	0.037832	-0.57451
APPL1	-0.23486	6.432585	-3.51567	0.000722	0.037832	-0.57602
TXLNG	-0.09616	5.608093	-3.51373	0.000726	0.037832	-0.58165
GOLPH3	-0.15014	9.802413	-3.51286	0.000728	0.037832	-0.58417
PDE8A	0.143832	7.419281	3.511584	0.000731	0.037832	-0.58788
BMP4	0.125178	7.499183	3.509818	0.000735	0.037832	-0.59301
EPN3	0.088829	5.304196	3.509678	0.000736	0.037832	-0.59342
AKT3	-0.08953	6.115786	-3.50695	0.000742	0.037877	-0.60132
PCYOX1L	0.15941	8.180091	3.505307	0.000746	0.037877	-0.60609
CYP4F2	-0.08861	6.45902	-3.50482	0.000748	0.037877	-0.60751
GPR4	0.070599	5.987335	3.504327	0.000749	0.037877	-0.60893
FST	0.081359	6.914799	3.501936	0.000755	0.038019	-0.61586
SPATA2L	-0.19628	6.969043	-3.49749	0.000766	0.038107	-0.62873
ZFC3H1	0.194122	8.914181	3.497003	0.000767	0.038107	-0.63015
PLCB3	-0.18212	6.764817	-3.49679	0.000767	0.038107	-0.63075
HMOX1	-0.29068	8.687179	-3.49602	0.000769	0.038107	-0.63299
CBR3	0.080047	6.22167	3.495051	0.000772	0.038107	-0.63579
RPS6KA2	-0.08644	6.411302	-3.49288	0.000777	0.038107	-0.64207
TP53TG1	-0.0848	5.998451	-3.49149	0.000781	0.038107	-0.64611
PAFAH2	0.081767	6.194093	3.490087	0.000784	0.038107	-0.65015
MXD3	-0.13269	7.401737	-3.48947	0.000786	0.038107	-0.65194
IMP3	0.14828	8.617638	3.489123	0.000787	0.038107	-0.65293
GNMT	0.113166	5.928385	3.4817	0.000806	0.038808	-0.67437
LOC10192	0.105612	6.866367	3.479824	0.000811	0.038808	-0.67978
RRP1B	-0.10678	7.511842	-3.47939	0.000812	0.038808	-0.68103
ATP2B4	-0.08977	7.869961	-3.47878	0.000814	0.038808	-0.6828
GRIP2	0.119908	7.317339	3.477102	0.000818	0.038872	-0.68762
PRDM4	0.078915	7.255926	3.474047	0.000826	0.038918	-0.69643
ERICH1	0.114545	6.300552	3.473372	0.000828	0.038918	-0.69837
RIPK2	0.100762	7.694494	3.472618	0.00083	0.038918	-0.70054
SDHA	0.104979	9.508454	3.472097	0.000831	0.038918	-0.70204
UBE3C	-0.16141	6.987987	-3.46918	0.000839	0.039141	-0.71044
NES	0.082234	6.678274	3.467571	0.000844	0.039199	-0.71507
OPHN1	-0.3378	9.175427	-3.46412	0.000853	0.039302	-0.72499
SST	0.090392	6.024149	3.463339	0.000855	0.039302	-0.72724
EFCC1	0.132171	6.603341	3.463224	0.000856	0.039302	-0.72757
TNFSF11	0.057616	5.235795	3.462214	0.000858	0.039302	-0.73047
OAZ3	0.066535	5.587586	3.459076	0.000867	0.039417	-0.73948
VSIG4	0.218019	7.558297	3.459061	0.000867	0.039417	-0.73953
COQ6	0.101419	6.721702	3.454873	0.000879	0.03976	-0.75155
RAB40B	-0.10885	5.615063	-3.45414	0.000881	0.03976	-0.75364
RNF11B	0.049361	4.991334	3.448887	0.000896	0.040295	-0.76871
TSPAN9	-0.08015	6.729772	-3.44734	0.000901	0.040352	-0.77314
N4BP3	-0.08263	5.810245	-3.44049	0.000921	0.04091	-0.79274
LOC10192	-0.10041	5.528252	-3.44014	0.000922	0.04091	-0.79375
AGBL3	-0.04759	4.727049	-3.43977	0.000923	0.04091	-0.79482
EGFR	0.054277	6.563734	3.435897	0.000934	0.041277	-0.80588
PKLR	-0.07505	6.143174	-3.43108	0.000949	0.041651	-0.81964
KCNK10	-0.15417	5.88226	-3.43091	0.000949	0.041651	-0.82011
LPO	0.094644	6.385714	3.425189	0.000967	0.042235	-0.83644
MPHOSPH	0.143161	7.237005	3.42441	0.00097	0.042235	-0.83866
CHMP7	-0.13191	7.616562	-3.42332	0.000973	0.042236	-0.84177

RPLP0	0.092612	12.55443	3.420803	0.000981	0.042432	-0.84894
MAP3K3	0.196165	7.890379	3.416842	0.000993	0.042827	-0.86021
CTSD	-0.33118	8.531537	-3.41549	0.000998	0.042865	-0.86406
CD320	0.139548	7.595893	3.41403	0.001002	0.042875	-0.86821
GSTK1	0.175889	10.23532	3.412719	0.001007	0.042875	-0.87194
SCD5	-0.43604	6.335566	-3.41223	0.001008	0.042875	-0.87334
IER2	-0.33803	10.91119	-3.4083	0.001021	0.043074	-0.8845
ORC3	0.130713	6.65092	3.407887	0.001022	0.043074	-0.88567
RWDD2B	0.125313	8.38008	3.407626	0.001023	0.043074	-0.88641
VPS39	-0.17226	7.868242	-3.40186	0.001042	0.043728	-0.90277
TRAP1	0.108083	8.27576	3.399866	0.001049	0.043862	-0.90843
EIF3E	0.130283	11.64947	3.398742	0.001053	0.043873	-0.91162
SOX21	0.110713	6.473123	3.39675	0.001059	0.044007	-0.91726
HLA-DRB6	0.091377	7.767164	3.395548	0.001063	0.044031	-0.92067
CASP10	0.071801	7.185454	3.392904	0.001072	0.044084	-0.92816
COPS3	0.168427	8.714074	3.392107	0.001075	0.044084	-0.93041
CTNNB1	-0.24985	8.494176	-3.39111	0.001079	0.044084	-0.93324
BRD7P3	0.098995	5.829786	3.391062	0.001079	0.044084	-0.93337
AKR1A1	0.131475	9.675051	3.388037	0.001089	0.044367	-0.94193
ACAP2	0.116857	7.420386	-3.38594	0.001097	0.04452	-0.94787
TRIM28	-0.24598	8.49513	-3.38358	0.001105	0.04459	-0.95452
SLMO1	0.085419	6.272938	3.383425	0.001105	0.04459	-0.95497
KRT15	-0.11572	5.517667	-3.37925	0.00112	0.044889	-0.96675
PRPSAP2	0.152089	7.96341	3.378941	0.001121	0.044889	-0.96763
44085	-0.07032	5.48524	-3.37802	0.001125	0.044889	-0.97024
CYP2W1	-0.17118	5.76618	-3.37731	0.001127	0.044889	-0.97223
FZR1	-0.10005	6.24441	-3.37589	0.001132	0.04495	-0.97623
ACTN1	-0.19109	8.505661	-3.37478	0.001136	0.044967	-0.97937
SLC35D2	-0.10074	6.662939	-3.3669	0.001165	0.04586	-1.00158
TBC1D10B	-0.1956	8.236085	-3.36579	0.001169	0.04586	-1.00469
TMEM161/	0.077891	8.400212	3.365628	0.00117	0.04586	-1.00515
ZNF91	0.261172	8.559731	3.363658	0.001177	0.046004	-1.01069
RUVBL2	0.136626	8.50302	3.36138	0.001186	0.046145	-1.0171
GNRHR	-0.0456	5.327403	-3.36022	0.00119	0.046145	-1.02037
CWC25	0.105184	7.83882	3.359763	0.001192	0.046145	-1.02164
BCOR	-0.08113	5.40141	-3.35738	0.001201	0.046276	-1.02834
BMP10	0.110247	6.577269	3.356923	0.001202	0.046276	-1.02962
APOBEC3F	-0.16004	5.763329	-3.3545	0.001212	0.046491	-1.03644
PTX3	-0.198	5.645913	-3.35167	0.001223	0.046766	-1.04436
ZNF654	0.178726	6.238139	3.347222	0.00124	0.047208	-1.05684
RAB32	0.126062	10.05577	3.346783	0.001242	0.047208	-1.05807
NKX6-1	-0.08644	5.476859	-3.34324	0.001256	0.047474	-1.06801
MYO9A	0.151624	7.147416	3.340972	0.001265	0.047474	-1.07435
ATP5A1	0.111831	11.91405	3.340552	0.001266	0.047474	-1.07552
DARS2	0.10582	7.344659	3.33989	0.001269	0.047474	-1.07737
ARSF	0.094028	6.93098	3.338652	0.001274	0.047474	-1.08084
ZNF771	0.051421	5.589234	3.338118	0.001276	0.047474	-1.08233
RP11-473I	-0.12642	5.906655	-3.33802	0.001277	0.047474	-1.08259
SNRPG	-0.15727	9.581101	-3.33743	0.001279	0.047474	-1.08426
OXA1L	0.125202	10.63012	3.336187	0.001284	0.04752	-1.08773
KEL	0.08629	6.932127	3.332095	0.001301	0.047996	-1.09917
RP11-457I	-0.07909	6.290435	-3.32911	0.001313	0.048308	-1.10749
C9orf116	0.056021	5.88177	3.328031	0.001317	0.048331	-1.11052
MYH14	-0.10573	6.364822	-3.32061	0.001349	0.04933	-1.1312
DDB2	0.143455	6.641909	3.319262	0.001354	0.049397	-1.13496
PNRC1	-0.15556	10.98918	-3.31703	0.001364	0.049552	-1.14118
GRK6	-0.17963	8.276263	-3.31642	0.001367	0.049552	-1.14287
RPN2	0.141196	10.36083	3.313962	0.001377	0.049623	-1.14972

KLHDC2	0.178655	7.986277	3.313209	0.00138	0.049623	-1.15181
TOB2	-0.17448	7.137355	-3.31141	0.001388	0.049623	-1.15682
RHBDF2	-0.20707	7.626083	-3.31114	0.001389	0.049623	-1.15756
COBLL1	0.063799	5.568212	3.309983	0.001395	0.049623	-1.16078
BTN2A1	0.118404	8.480865	3.309447	0.001397	0.049623	-1.16227
ZNF33B	0.115434	6.892873	3.309092	0.001398	0.049623	-1.16326
FAM222B	0.091907	7.189638	3.307702	0.001405	0.049623	-1.16712
CAPZA1	-0.15154	10.98012	-3.3075	0.001405	0.049623	-1.16767
PAFAH1B2	0.091207	5.731233	3.306546	0.00141	0.049623	-1.17033
ONECUT2	0.093412	6.245917	3.306012	0.001412	0.049623	-1.17182
