# Supplementary material for: Identification of Potential Osteoporosis miRNA Biomarkers Using Bioinformatics Approaches
Source: Comput Math Methods Med. 2021 Nov 2;2021:3562942. doi: 10.1155/2021/3562942 (PMC8579105; doi:10.1155/2021/3562942)
Supplement: Supplementary 2 — Supplementary Table 2: primer sequences of qRT-PCR. [file 3562942.f2.pdf]

miRNAs	primers
miR-1271-5p	F: 5′-CAGCACTTGGCACCTAGCA-3′
	R: 5′-TATGGTTGTTCTCCTCTCTGTCTC-3′
miR-132-3p	F: 5′-GCGCGCGTAACAGTCTACAGC-3′
	R: 5′-GTCGTATCCAGTGCAGGGTCC-3′
miR-135a-5p	F: 5′-AACCCTGCTCGCAGTATTTGAG-3′
	R: 5′-GCGGCAGTATGGCTTTTTATTCC-3′
miR-135b-5p	F: 5′-GGTATGGCTTTTCATTCCT-3′
	R: 5′-GCGAGCACAGAATTAATACGAC-3′
miR-153-3p	F: 5′-ACACTCCAGCTGGGTTGCATAGTCACAAA-3′
	R: 5′-CAGTGCGTGTCGTGGAGT-3′
miR-15a-5p	F: 5′-CTAAAGAGGCGGCAGGTCCCTC-3′
	R: 5'-CAAGATGACCTTCCAGTGAC-3'
miR-15b-5p	F: 5′-TAGCAGCACATCATGGTTTACA-3′
	R: 5′-TGCGTGTCGTGGAGTC-3′
miR-182-5p	F: 5′-TTTGGCAATGGTAGAACTCACACT-3′
	R: 5′-TGGTGCAGGGTCCGAGGTAT-3′
miR-223-3p	F: 5'-GTGCAGGGTCCGAGGT-3'
	R: 5'-CGGGCTGTCAGTTTGTCA-3'
miR-27a-3p	F: 5′-GGCTTAGCTGCTTGTGA-3′
	R: 5′-GAACATGTCTGCGTATCTC-3′
miR-27b-3p	F: 5′-TCTAGATG CTCC TCCAGAAACCGTGG-3′
	R: 5′-GAATTCCCTTTCAGACAGATGTCCGA-3′
miR-370-3p	F: 5′-GCCTGCTGGGGTGGAACCTGGT-3′
	R: 5′-GCAGGGTCCGAGGTATTC-3′
miR-486-5p	F: 5′-CTCGCTTCGGCAGCACA-3′
	R: 5′-ACGCTTCACGAATTTGCGT-3′
miR-9-5p	F: 5′-GGGTCTTTGGTTATCTAGC-3′
	R: 5′- TGCGTGTCGTGGAGTC-3′
miR-96-5p	F: 5′-ATGCTTTCTCAACTTGTTGG-3′
	R: 5′-TCACCG CTCTTGGCCGTCACA-3′
U6	F: 5\`-GCTCGCTTCGGCAGCACA-3
	R: 5\`-GAGGTATTCGCACCAGAGGA-3\`
