# Supplementary material for: Identification of Potential Osteoporosis miRNA Biomarkers Using Bioinformatics Approaches
Source: Comput Math Methods Med. 2021 Nov 2;2021:3562942. doi: 10.1155/2021/3562942 (PMC8579105; doi:10.1155/2021/3562942)
Supplement: Supplementary 3 — Supplementary Table 3: miRNAs regulating genes in key functional subsets. [file 3562942.f3.pdf]

miRNA	mRNA
hsa-let-7a-5p	WNT1
hsa-let-7e-5p	WNT1
hsa-miR-1271-5p	FOXO1
hsa-miR-132-3p	FOXO1
hsa-miR-133b	EGFR
hsa-miR-135a-5p	FOXO1
hsa-miR-135b-5p	FOXO1
hsa-miR-137	EGFR
hsa-miR-137	MITF
hsa-miR-139-5p	JUN
hsa-miR-148a-3p	WNT1
hsa-miR-148b-3p	WNT1
hsa-miR-152-3p	WNT1
hsa-miR-153-3p	FOXO1
hsa-miR-15a-5p	AKT3
hsa-miR-15a-5p	FOXO1
hsa-miR-15b-5p	AKT3
hsa-miR-15b-5p	FOXO1
hsa-miR-16-5p	AKT3
hsa-miR-182-5p	FOXO1
hsa-miR-182-5p	MITF
hsa-miR-195-5p	AKT3
hsa-miR-200b-3p	JUN
hsa-miR-200c-3p	JUN
hsa-miR-20b-5p	AKT3
hsa-miR-216a-5p	MITF
hsa-miR-216b-5p	JUN
hsa-miR-218-5p	MITF
hsa-miR-22-3p	WNT1
hsa-miR-223-3p	FOXO1
hsa-miR-27a-3p	FOXO1
hsa-miR-27b-3p	FOXO1
hsa-miR-29a-3p	AKT3
hsa-miR-29b-3p	AKT3
hsa-miR-29c-3p	AKT3
hsa-miR-302b-3p	EGFR
hsa-miR-340-5p	MITF
hsa-miR-34a-5p	WNT1
hsa-miR-370-3p	FOXO1
hsa-miR-381-3p	NFKBIA
hsa-miR-424-5p	AKT3
hsa-miR-429	JUN
hsa-miR-486-5p	FOXO1
hsa-miR-497-5p	AKT3
hsa-miR-503-5p	AKT3
hsa-miR-519d-3p	AKT3
hsa-miR-520b	EGFR
hsa-miR-520e	EGFR
hsa-miR-6838-5p	AKT3
hsa-miR-7-5p	AKT3
hsa-miR-7-5p	EGFR
hsa-miR-875-5p	EGFR
hsa-miR-9-5p	FOXO1
hsa-miR-96-5p	FOXO1
hsa-miR-96-5p	MITF
